# Effects of supplementation with main coffee components including caffeine and/or chlorogenic acid on hepatic, metabolic, and inflammatory indices in patients with non-alcoholic fatty liver disease and type 2 diabetes: a randomized, double-blind, placebo-controlled, clinical trial

**DOI:** 10.1186/s12937-021-00694-5

**Published:** 2021-04-10

**Authors:** Asieh Mansour, Mohammad Reza Mohajeri-Tehrani, Majid Samadi, Mostafa Qorbani, Shahin Merat, Hossein Adibi, Hossein Poustchi, Azita Hekmatdoost

**Affiliations:** 1 Department of Clinical Nutrition & Dietetics, Faculty of Nutrition Sciences and Food Technology, National Nutrition and Food Technology Research Institute, Shahid Beheshti University of Medical Sciences, Tehran, Iran; 2 Endocrinology and Metabolism Research Center, Endocrinology and Metabolism Clinical Sciences Institute, Tehran University of Medical Sciences, Tehran, Iran; 3 Radiology Department, Shariati Hospital, Tehran University of Medical Sciences, Tehran, Iran; 4 Non-communicable Diseases Research Center, Alborz University of Medical Sciences, Karaj, Iran; 5 Chronic Diseases Research Center, Endocrinology and Metabolism Population Sciences Institute, Endocrinology and Metabolism Research Institute, Tehran University of Medical Sciences, Tehran, Iran; 6 Liver and Pancreatobiliary Diseases Research Center, Digestive Diseases Research Institute, Tehran University of Medical Sciences, Tehran, Iran; 7 Diabetes Research Center, Endocrinology and Metabolism Clinical Sciences Institute, Tehran University of Medical Sciences, Tehran, Iran

**Keywords:** Coffee, Caffeine, chlorogenic acid, Type 2 diabetes, Non-alcoholic fatty liver disease, Clinical trial

## Abstract

**Background:**

Non-alcoholic fatty liver disease (NAFLD) is much more frequent and more severe, including *cirrhosis,* hepatocellular carcinoma in patients with type 2 diabetes. Coffee is a complex beverage with hundreds of compounds whereas caffeine and chlorogenic acid are the most abundant bioactive compounds. The published epidemiological data demonstrating beneficial associations between all categories of coffee exposure and ranges of liver outcomes are rapidly growing; however, the main contributors and cause-effect relationships have not yet been elucidated. To address existing knowledge gaps, we sought to determine the efficacy and safety of 6 months chlorogenic acid and/or caffeine supplementation in patients with type 2 diabetes affected by NAFLD.

**Methods:**

This trial was carried out at two Diabetes Centers to assess the effects of supplementation with daily doses of 200 mg chlorogenic acid, 200 mg caffeine, 200 mg chlorogenic acid plus 200 mg caffeine or placebo (starch) in patients with type 2 diabetes and NAFLD. The primary endpoint was reduction of hepatic fat and stiffness measured by FibroScan, and changes in serum hepatic enzymes and cytokeratin − 18 (CK-18) levels. Secondary endpoints were improvements in metabolic (including fasting glucose, homeostasis model assessment-estimated insulin resistance (HOMA-IR), hemoglobin A1c (HBA1C), C-peptide, insulin and lipid profiles) and inflammatory (including nuclear factor k-B (NF-KB), tumor necrosis factor (TNF-α), high sensitive- C reactive protein(hs-CRP)) parameters from baseline to the end of treatment.

**Results:**

Neither chlorogenic acid nor caffeine was superior to placebo in attenuation of the hepatic fat and stiffness and other hepatic outcomes in patients with diabetes and NAFLD. Except for the lower level of total cholesterol in caffeine group (*p* = 0.04), and higher level of insulin in chlorogenic acid plus caffeine group (*p* = 0.01) compared with placebo, there were no significant differences among the treatment groups.

**Conclusion:**

These findings do not recommend caffeine and/or chlorogenic acid to treat NAFLD in type 2 diabetes patients.

**Trial registration:**

IRCT201707024010N21. Registered 14 September 2017.

## Introduction

Patients with type 2 diabetes have a risk of developing serious liver related complications including non-alcoholic fatty liver disease (NAFLD), hepatic cirrhosis, and hepatocellular carcinoma that is two to four times higher than those without diabetes [1].

Observational studies in different populations have suggested that coffee consumption is associated with a reduced risk of NAFLD [2, 3], liver fibrosis [4], type 2 diabetes [5], and hepatocellular carcinoma [6]. It is well established that coffee is a mixture of various chemical compounds; among the identified constituents, caffeine, chlorogenic acids, and two diterpenes (cafestol and kahweol) are the main compounds [7]. Caffeine is thought to prevent or reverse hepatic fibrosis by several mechanisms including acting as an A2A receptor antagonist which affect the activation of hepatic stellate cells [8]. Recent meta -analysis suggests that this association may be explained by caffeine and not by the decaffeinated coffee. Caffeine is thought to inhibit the proliferation of hepatocellular carcinoma cell [9]. Data from the National Health and Nutrition Examination Surveys (NHANES) that collected between 2001 and 2008 indicated caffeine intake as one of the five independent predictors of NAFLD, suggesting that caffeine may have a potential protective effect [10]. However, a meta- analysis has shown that regular coffee caffeine consumption, but not total caffeine intake, is associated with a lower levels of fibrosis severity [7]. This finding might suggest other ingredients in regular coffee, rather than caffeine, could be responsible for its antifibrotic effects [11]. Chlorogenic acid (a major polyphenol in coffee) plays an important role on modulating glucose intolerance and hyperlipidemia [12], might also play an important role in hepatoprotection. Diterpenes are found in coffee as esters of fatty acid and are known to have a cholesterol raising properties [13]. It is remained unknown what bioactive component in coffee contributes its part more significantly for the reported effects [14].

Although recent meta-analysis of observational studies have shown the beneficial association of coffee consumption with risk reduction of NAFLD and liver fibrosis [15], observational studies can only show association, but cannot provide evidence of causal relationship. The relationship between coffee consumption and liver fibrosis might be due to other factors in frequent coffee drinkers behavior. The hepatoprotective benefits of coffee have not been studied in randomized controlled trials. So, it could not be included in evidence based treatment recommendations. Thus, clinical trials are necessary to find a possible cause-effect relationship, while finding the main effective components of coffee, and their mechanism of action. Therefore, we performed this randomized clinical trial to assess the efficacy and safety of two main components of coffee including caffeine and/or chlorogenic acid at a dose of 200 mg daily in patients who had type 2 diabetes accompanied with NAFLD.

## Methods

Enrollment occurred at two clinics of diabetes in Endocrinology and Metabolism Research Center (Tehran University of Medical Sciences, Tehran, Iran), from September 29, 2017 through March 19, 2018 and the final date of follow up was October 10, 2018. The protocol (available at nimad.ac.ir) was approved by the National Institute for Medical Research Developments Ethics Committee (IR.NIMAD.REC.1396.028), and all participants provided written informed consent. Capsules containing the caffeine and/ or chlorogenic acid and matching placeboes were provided by Arjuna Natural Extracts, India.

### Study population

Men and women aged 30–53 years with type 2 diabetes (based on 1999 World Health Organization (WHO) criteria) and NAFLD (controlled attenuation parameter (CAP) score of 260 or more) were eligible for enrollment. Exclusion criteria were current use of insulin, current pregnancy or breastfeeding, alcohol intake as defined by an average daily consumption of alcohol > 20 g/day within the previous year, evidence of other forms of liver diseases (such as viral hepatitis, autoimmune hepatitis, etc.), evidence of cirrhosis (according to transient elastography (TE) exam and biochemical profile), current supplementation with vitamin E and other antioxidants, treatment with Milk thistle (*Silybummarianum*) (to reduce the effects of confounding factors related to treatment strategies) in the past 6 month or during the study, a history of thyroid, kidney, and mental disease, heart failure, and cancer. Subjects were asked to bring their current medications at the time of screening, and they were checked by the investigator.

### Study procedures

We identified potential patients treated with stable dose of oral anti-hyperglycemic agents and recruited them to fill out a screening questionnaire. Those who answered the questionnaire indicating that they are willing and eligible to participate in the trial, entered in the diagnosis for NAFLD phase, during which, we invited them to undergo abdominal ultrasonography. Patients who had hepatic steatosis of grade 2 or more were examined by TE. Patients who remained eligible (a CAP score level of at least 260 dB/m but Liver stiffness measurements (LSM) levels less than 14 kPa) were randomly assigned by a double-blind fashion using random blocked randomization in a 1:1:1:1 ratio to receive 2 capsules containing either 200 mg of chlorogenic acid plus 200 mg of caffeine (about 2 cups of coffee), or 200 mg caffeine plus placebo (starch), or 200 mg chlorogenic acid plus placebo, or two matching placebo capsules daily for 6 months. All of investigators involved in this study were blinded to the treatments groups until final analysis. Study visits were conducted at baseline and at 6, 12, 18, 24 weeks after initiation of the trial. At each visit, information was gathered regarding adverse events and adherence to study. At the baseline and after 24 weeks of follow up, we collected blood samples, along with anthropometric, blood pressure, and non-invasive markers of hepatic steatosis and fibrosis measurements.

### Outcomes

LSM expressed in kilopascals (kPa) and CAP score expressed in dB/m were assessed using FibroScan (EchoSense, Paris, France) in participants fasted for at least 3 h according to the manufacturer’s guideline.

Nutritionist IV software (First Data Bank, The Hearst Corporation, San Bruno, CA) was used for the analysis of 24 h dietary recalls (5 weekdays and 2 weekend). In addition, physical activity was estimated using the international physical activity questionnaire (IPAQ) by multiplying MET (Metabolic Equivalent of Task) and time spent in each activity [16]. To assess potential changes in daily food consumption and physical activity during the 6-month intervention, participants kept a 7-day food record and a record of their physical activity at baseline and at month 6 of the intervention. All participants were asked to maintain their usual dietary intake and habitual physical activity for the duration of trial.

Resting energy expenditure was measured after 12 h fasting, using Fitmate Calorimeter (Cosmed, Rome, Italy), while the patients were lied down. Body composition was assessed using body impedance analyzer (BIA) (Tanita, Ilinois, USA).

Blood samplings were performed early in the morning after an overnight fast of 10-12 h. The blood collection from participants, blood withdrawal and blood analyses were done by trained and experienced laboratory technicians in laboratory of Endocrinology and Metabolism Research Institute under suitable conditions. Serum liver enzymes (alanine transaminase (ALT), aspartate transaminase (AST), and gamma-glutamyl transferase (GGT)), total cholesterol (TC), high-density lipoprotein cholesterol (HDL), triglycerides (TG), creatinine (Cr) and high sensitive- C reactive protein (hs-CRP) measured by using ELISA kit (Roche, Germany). Low-density lipoprotein cholesterol (LDL) values were calculated using the Friedewald formula. Hemoglobin A1c (HbA1c) levels were assessed by a high performance liquid chromatography analyzer (Tosoh, Tokyo, Japan). Fasting blood glucose levels were measured using the glucose oxidase method on an auto analyzer (Cobas c 311, Roche Dignostics, Risch-Rotkreuz, Switzerland). Serum insulin, C-peptide and thyroid stimulating hormone (TSH) levels were measured by using ELISA kit (Monobind Inc. Lake Forest, California, USA). Homeostasis model assessment-estimated insulin resistance (HOMA-IR) score was used to determine the degree of insulin resistance using the following formula: HOMA-IR = Fasting insulin (μU/mL) × Fasting glucose (mmol/L)/22.5. Serum concentrations of total antioxidant capacity (TAC), adiponectin, and cytokeratin-18 (CK-18) were measured using ELISA method (ZellBio GmbH, Ulm, Germany). Serum tumor necrosis factor (TNF)-α was measured by ELISA (Diaclone, France). Nuclear factor k-B (NF-kB) levels was assessed in peripheral blood mononuclear cells (PBMCs) nuclear extracts using an ELISA kit (ZellBio GmbH, Ulm, Germany) according to the manufacturer’s instructions.

### Statistical analysis

The sample size was calculated for the CAP score, which was based on detection of a 10 unit (dB/m) difference in the mean CAP score with a power of 80% (β = 20%), yielding a sample size of 21 for each group. Given the probability of samples loss, twenty five patients in each group were considered [17].

All statistical analyses were performed using SPSS version 16.0 (SPSS Inc., Chicago, IL, USA) on an intention-to-treat basis. Quantitative variables are presented as the mean ± SD. One-way ANOVA were used to examine the consistency of study groups. The Kolmogorov–Smirnov test was used to determine normality of distribution of the examined variables. Non-normally distributed variables were log transformed. Repeated-measures of ANOVA were used after intervention for assessment of differences between the treatment groups with adjusting for baseline insulin and the baseline value of the outcome being analyzed. Tukey significance difference test was used for post hoc multiple comparisons. Enumeration data were analyzed by the Chi-squared test. Two-tailed *P* < 0.05 was considered significant.

## Results

**Fig. 1:**
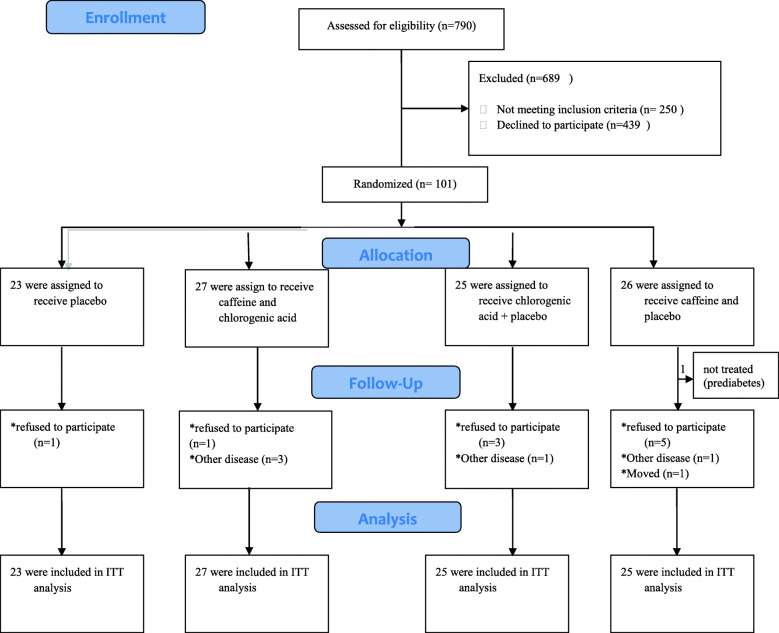
trial profile. ITT intention to treat

From September 2017 through March 2018, a total of 101 patients underwent randomization to receive placebo (*n* = 23), caffeine plus chlorogenic acid (*n* = 27), chlorogenic acid alone (*n* = 25), or caffeine alone (*n* = 26); however, we excluded one patient from caffeine alone group who had a diagnosis of prediabetes. Totally, a hundred participants comprised the intention- to- treat population, of whom 84 (84%) completed the study (Fig. 1). There were no meaningful imbalance in demographic characteristics or use of ongoing medications (Table 1), or dietary intake (Table 2), clinical and laboratory data with the exception of insulin levels (*p* = 0.009)  (data not shown). The mean age of all patients was 44.57 ± 5.65 years, with a mean diabetes duration of 4.57 ± 4.23 years and the majority of them were male (68%). There was no significant difference in dietary intakes among the groups before and after the intervention.

**Table 1:** Demographic characteristics, anthropometrics and use of ongoing medications of study population according to group of treatment at baseline

Characteristic	Placebo (*n* = 23)	Cholrogenic acid+ caffeine (*n* = 27)	Cholrogenic acid (*n* = 25)	Caffeine (*n* = 25)	Pvalue
Age (yr)	44.17 ± 4.98	43.59 ± 5.51	44.88 ± 6.91	45.68 ± 5.07	0.58^¥^
Male sex, No (%)	15(65.2)	17(63)	19(76)	17(68)	0.76*
Known duration of diabetes (yr)	4.05 ± 4.49	3.84 ± 3.65	4.37 ± 3.25	5.97 ± 5.27	0.22^¥^
Number of current smokers (%)	4(17.4)	7(25.9)	3(12)	4(16)	0.6*
Height (cm)	170.91 ± 9.83	170.13 ± 9.74	172.48 ± 8.04	170.32 ± 9.76	0.8^¥^
BMI (kg/m^2^)	30.6 ± 3.1	31.26 ± 4.76	30.84 ± 3.28	29.59 ± 3.4	0.45^¥^
Fat mass (kg)	27.23 ± 7.16	28.9 ± 10.31	26.47 ± 6.94	24.74 ± 8.27	0.35^¥^
FFM (kg)	62.1 ± 11.29	61.48 ± 12.33	64.54 ± 10.95	60.58 ± 9.59	0.67^¥^
Waist circumferences (cm)	107.48 ± 9.12	108.91 ± 10.9	110.26 ± 9.5	104.6 ± 6.87	0.17^¥^
REE (kcal)	1833.37 ± 395.42	1859.59 ± 381.41	1865 ± 339.25	1750.09 ± 314.37	0.68^¥^
Physical activity (METs h/day)	30.52 ± 5.19	30.54 ± 4.08	32.18 ± 4.39	31.75 ± 3.94	0.43^¥^
Medications, n(%)					
Metformin	20(87)	23(85)	23(92)	21(84)	0.84*
DPP4 Inhibitors	6(26.1)	6(22.2)	4(16)	11(44)	0.14*
Sulfonamide	5(21.7)	9(33.3)	10(40)	9(36)	0.57*
Beta blocker+ABRS	6(26.1)	6(22.2)	3(12)	4(16)	0.59*
Statin	6(26)	10(37)	10(40)	11(44)	0.61*
Other lipid lowering gents	0	3(11.1)	2(8)	1(4)	0.38*
Aspirin	3(13)	2(7.4)	2(8)	4(16)	0.72*

^¥^ One-way ANOVA

*chi- square

Two-tailed *P* < 0.05 was considered significant

Each value represents mean ± SD except for gender, smoking, ongoing use of medication n(%). BMI body mass index, FFM fat free mass, REE resting energy expenditure, METs Metabolic Equivalents, DPP4 Dipeptidyl Peptidase 4, ARBS Angiotensin II receptor blockers

**Table 2:** Energy and nutrients intake among treatment groups at baseline and after 6 months

Characteristic	Placebo (*n* = 23)	Cholrogenic acid+ caffeine (*n* = 27)	Cholrogenic acid (*n* = 25)	Caffeine (*n* = 25)	Pvalue
Energy intake (kcal)					
Baseline	2536.16 ± 403.88	2603.12 ± 595.55	2626 ± 485.68	2855.62 ± 445.29	0.32^¥^
6 months	2313.42 ± 441.25	2459.41 ± 432.01	2412.63 ± 402.49	2385.50 ± 495.96	0.08*
Protein (g)					
Baseline	79 ± 23.3	85.61 ± 31.94	80.35 ± 28.26	93.46 ± 6.56	0.5^¥^
6 months	70.24 ± 20.47	83.89 ± 20.65	75.41 ± 18.99	82.56 ± 30	0.6*
Fat(g)					
Baseline	120.14 ± 18.23	121.38 ± 28.22	105.65 ± 23.48	124.9 ± 18.04	0.13^¥^
6 months	108.32 ± 27.22	98.77 ± 34.69	100.99 ± 17.69	107.81 ± 25.41	0.36*
Carbohydrate (g)					
Baseline	293.9 ± 71.37	302.16 ± 89.07	351.04 ± 53.98	350.55 ± 85.39	0.1^¥^
6 months	237.22 ± 66.01	320.1 ± 95.12	309.87 ± 70.03	283.15 ± 73.11	0.15*
Caffeine (mg)**					
Baseline	79.75 ± 42.94	97.11 ± 51.08	136..55 ± 105.74	130.68 ± 82.77	0.16^¥^
6 months	131.78 ± 98.35	117.28 ± 82.02	110.32 ± 90.87	130.51 ± 125.67	0.37*

Data are mean ± SD

^¥^ One-way ANOVA

*Time X Treatment interaction according to two-way repeated measures ANOVA

**The amount of caffeine consumed in foods and *beverages*

Two-tailed P < 0.05 was considered significant

**Table 3:** Changed in hepatic indicators, metabolic profile, inflammatory and total antioxidant levels vs. placebo

Characteristic	Adjusted treatment differences (95%CI;p)		
	Chlorogenic acid+ caffeine vs placebo	Chlorogenic acid vs placebo	Caffeine vs placebo
Fibro score (KPa)	0.43(− 0.46 to 1.32; *p* = 0.3)	0.66 (− 0.21 to 1.53; *P* = 0.09)	0.24 (− 0.62 to1.12;*P* = 0.57)
CAP score (dB/m)	12.06(−7.3 to 31.46;*P* = 0.26)	13.07(−5.6 to 32.13; P = 0.2)	−7.45 (−26.48 to 11.57; *P* = 0.31)
CK-18 fragments (U/L)	−0.00(− 0.22 to 0.21; *P* = 0.42)	0.04(− 0.16 to 0.25; *P* = 0.85)	− 0.13(− 0.33 to 0.08;*P* = 0.17)
AST(U/L)	2.11(− 2.7 to 6.97;*P* = 0.37)	3.07(− 1.71 to 7.85; *P* = 0.13)	− 0.43 (− 5.19 to 4.34; *P* = 0.87)
ALT(U/L)	1.9(− 2.45 to 6.23;*P* = 0.62)	2.4(− 1.8 to 6.7;*P* = 0.17)	−0.12(− 4.4 to 4.13;*P* = 0.79)
GGT(U/L)	0.78(− 16.68 to 15.13;*P* = 0.94)	3.48(− 12.13to 19.1;*P* = 0.41)	8.55(− 7.05 to 24.16;*P* = 0.35)
Fasting glucose (mg/dl)	− 1.76(− 30.08 to 26.46; *P* = 0.97)	4.9(− 22.9 to 32.7; *P* = 0.74)	−2.99(− 30.77 to 24.78; *P* = 0.86)
HbA1C (%)	−0.25(− 1.21 to 0.71; *P* = 0.66)	0.04 (− 0.9 to 1;*P* = 0.98)	−0.55(− 1.5 to 0.39; *P* = 0.18)
Insulin (uIU/ml)	3.3(− 1.3 to 6.7; *P* = 0.01)	0.2(− 3.28 to 3.7; *P* = 0.67)	− 0.16(− 3.66 to 3.32; *P* = 0.71)
C-peptide (ng/ml)	0.04(− 0.27 to 0.36; *P* = 0.96)	− 0.03(− 0.35 to 0.28; *P* = 0.84)	−0.06(− 0.37 to 0.26; *P* = 0.51)
HOMA-IR score	0.4(− 0.62 to 1.42; *P* = 0.69)	0.01(− 0.99 to 1.01; *P* = 0.78)	−0.01(− 1.02 to 0.99; *P* = 0.91)
TG (mg/dl)	−10.56(− 140.54 to 119.42; *P* = 0.89)	46.17(−81.45 to 173.79; *P* = 0.65)	− 62.99(− 190.50 to 64.53; *P* = 0.17)
Cholesterol (mg/dl)	−9.43(− 29.7 to 10.83;P = 0.5)	−5.07(− 24.97 to14.82;*P* = 0.7)	−20.66(− 40 .55 to − 0.78; *P* = 0.04)
HDL (mg/dl)	−0.94(− 5.83 to 3.95; *P* = 0.79)	0.27(− 4.5 to 5.07; *P* = 0.89)	1.62(− 3.18 to 6.41; *P* = 0.52)
LDL (mg/dl)	−5.77(− 21.3 to 9.76; *P* = 0.49)	−5.58(20.98 to 9.81;*P* = 0.48)	−11.93(− 27.16 to 3.3; *P* = 0.12)
hs-CRP (mg/dl)	0.73(−2.61 to 4.08; *P* = 0.6)	− 1.64(− 4.93 to 1.64;*P* = 0.33)	0.02(− 3.25 to 3.31; *P* = 0.99)
TNF-α (pg/ml)	0.5(− 8.27 to 9.2; *P* = 0.83)	−4.96(− 13.33 to 3.4;*P* = 0.24)	−1.59(− 10.02 to 6.84; *P* = 0.96)
NF-KB (ng/mg protein)	−0.16(− 1.08 to 0.77; *P* = 0.68)	0.23(− 0.67 to 1.14; *P* = 0.9)	−0.23(− 0.93 to 0.88;*P* = 0.8)
Adiponectin (mg/L)	0.29(− 1.82 to 2.4; *P* = 0.92)	0.07(− 1.95 to 2.1; *P* = 0.86)	−2.43(− 4.47 to − 0.4;*P* = 0.06)
TAC (mmol/L)	0.00(− 0.04 to 0.05;*P* = 0.69)	−0.00(− 0.05 to 0.04; *P* = 0.92)	0.00(− 0.04 to 0.04;*P* = 0.79)
Cr (mg/dl)	−0.00(− 0.1 to 0.09; *P* = 0.84)	0.02(− 0.07 to 0.11; *P* = 0.63)	0.07(− 0.02 to 0.17;*P* = 0.12)
TSH (mIU/L)	−0.11(− 0.9 to 0.66; *P* = 0.32)	0.24(− 0.52 to 1.01; *P* = 0.85)	0.06(− 0.71 to 0.82;*P* = 0.75)
Weight (kg)	0.63(−6.33 to 7.59;*p* = 0.51)	2.36(−4.73 to 9.45;*p* = 0.63)	−3.39(− 10.49 to3.7;*p* = 0.38)
Systolic blood pressure (mmHg)	0.26(−6.93 to 7.45;*P* = 0.84)	1.02(− 6.04 to 8.08; *P* = 0.68)	−1.41(− 8.47 to 5.65; *P* = 0.83)
Diastolic blood pressure (mmHg)	− 1.69(− 6.83 to 3.46; *P* = 0.59)	−1.27(− 6.32 to 3.78; *P* = 0.77)	−1.2(− 6.25 to 3.84; *P* = 0.83)

^a^Tukey significance difference test was used for post hoc multiple comparisons. Two-tailed *P* < 0.05 was considered significant

Data not conforming to a normal distribution were log-transformed prior to parametric analysis. CAP -Score controlled attenuation parameter Score, CK-18 cytokeratin-18, AST aspartate aminotransferase, ALT alanine aminotransferase, GGT gamma-glutamyltransferase, HbA1c hemoglobinA1c, HOMA-IR homeostasis model assessment insulin resistance, TG Triglyceride, HDL *high-density lipoprotein, LDL low-density lipoprotein*, hs-CRP *high-Sensitivity C-Reactive Protein,* TNF-α *tumor necrosis factor-α*, *NF-κB nuclear factor kappa B*, TAC total antioxidant capacity, Cr creatinine, TSH thyroid stimulating hormone

Table 3 shows the differences in hepatic indicators, metabolic profile, inflammatory and total antioxidant levels among treatment groups at the end of study adjusted for the baseline values and insulin levels. CAP scores and LSM were not significantly different among the groups. Similarly, no significant differences between groups were evident in other hepatic indicators including AST, ALT, GGT, Ck-18 levels (*p* > 0.05). Except for increase in fasting insulin (mean differences 3.3 μIU/ml [95%CI, − 1.3 to 6.7]; *p* = 0.01), in chlorogenic acid plus caffeine compared with placebo, none of the other supplementations were significantly associated with changes in fasting glucose, HOMA-IR, HBA1C or C-peptide compared with placebo. Supplementation with caffeine significantly decreased cholesterol levels as compared with placebo (mean differences − 20.66 mg/dl [95%CI, − 40.55 to − 0.78]; *p* = 0.04), whereas LDL- cholesterol, HDL- cholesterol, and TG were not affected by any other groups of treatments. No other significant differences among the four groups were found in those treated with caffeine or/ and chlorogenic acid compared with placebo regarding all other biomarkers (NF-KB, TNF-α, hs-CRP, TAC, adiponectin, Cr and TSH). Moreover, systolic and diastolic blood pressure changes did not differ significantly among the groups. No side effects were reported during the study period.

## Discussion

This is the first randomized, double-blind, placebo-controlled trial of chlorogenic acid and/ or caffeine supplementation performed in type 2 diabetes patients with NAFLD. This trial showed that an intervention based on main components of coffee has no significant effect on hepatic fat content and fibrosis in type 2 diabetic patients affected by NAFLD. Six months supplementation with caffeine and/or chlorogenic acid did not improve either hepatic enzymes (ALT, AST, and GGT) or the serum levels of CK-18 as a specific biomarker for liver injury. As we specifically aimed to investigate the effects of coffee main constitutes on NAFLD in patients with diabetes, we also tested the hypothesis of an involvement of chlorogenic acid and/ or caffeine in modulating insulin resistance (HOMA-IR), metabolic and glycemic control (fasting glucose, HBA1C) in these subjects. Our results have shown that except for increase in insulin in chlorogenic acid plus caffeine group, and decrease in cholesterol in caffeine group, none of these parameters significantly changed after 6 months intervention in comparison with the placebo group. Likewise, the supplementation did not induce any specific effect on thyroid and renal function as measured by TSH and Cr levels, respectively. Similarly, neither inflammatory indictors nor antioxidant capacity changes were significantly different between active treated groups and placebo after 6 months.

Decaffeinated and regular coffee consumption have been investigated in prevention and treatment of NAFLD with varying results in many large observational studies; most of which reported that coffee has beneficial impact on liver; however rarely, it was the main target of interest or studied in clinical trials [3, 18]. In pre -clinical studies, caffeine seems to be able to exert inhibitory effects on fibrogenesis and block the progression of liver steatosis through interruption in transforming growth factor-β (TGF-β) signaling, inhibition of stellate cells and by its antioxidant and anti-inflammatory properties [19]. However, contrary to these hypotheses, we observed that caffeine and / or chlorogenic acid did not significantly affect liver outcomes after 6 months supplementation. Coffee contains other substances (e.g. melanoidins, niacin, diterphenoic alcohols) in addition to caffeine and chlorogenic acid that could exert a protective effect on a range of liver diseases [18, 20]. In the present study, the amount of caffeine or chlorogenic acid ingested by the subjects was 200 mg/day, which equates to the amount of caffeine found in 2 cups of coffee, which might not be enough to show any effect. Observational studies showed that drinking more than 3 cups coffee per day is protective against fatty liver [18, 21]. Since increase in blood pressure with regular coffee or caffeine intake have been observed [22], we were not ensured that most of our patients would easily tolerate upper doses of coffee (above 200 mg/d). However, this study did not provide support for the adverse relationship between coffee and blood pressure and even led to non-significant 1.2–1.69 mmHg decrease in diastolic blood pressure. A meta- analysis of clinical trials and prospective cohort studies’ results are in consistent with our study, suggesting no evidence of any relationship between coffee consumption and hypertension [23]. Zhang et al. described an inverse J-shaped association between intake of this beverage and hypertension risk; so that one to three cups coffee/day intake compared with less than one cup/day could increase the risk, while protective effects were observed at higher intakes [24]. Another meta-analyses concluded a beneficial effect of coffee on blood pressure [25, 26]. It is important to note that sex, race, and lifestyle factors (e.g., smoking) have been considered as factors involved in the different shape of relationship between coffee use and blood pressure [22]. In addition, inter -individual differences mainly due to the variation in caffeine metabolism related genes including CYP1A2 (slow caffeine metabolizers vs. rapid metabolizers) may partially explain conflicting results [24]. As we aimed specifically to assess the efficacy of chlorogenic acid and/ or caffeine supplementation on liver steatosis in type 2 diabetes patients, our sample consisted of patients attending to our diabetes outpatients’ center for routine diabetes care. So, this setting led to recruitment of cases with mild degree of NAFLD rather than those with advanced liver disease referring to hepatology clinics. The results from Bambha et al. study showed that in those with less insulin resistance, high coffee consumption was inversely associated with advanced fibrosis, as compared with those with greater insulin resistance. So, it could speculate less benefit of coffee consumption on liver fibrosis treatment in patients with diabetes who have insulin resistance [14]. Indeed, we cannot rule out the effect of coffee components in NAFLD patients with greater fibrosis or patients with less insulin resistance or non-diabetic patients. The lack of significant effects of coffee on liver outcomes are consistent with the non-significant effects of coffee on antioxidant and inflammatory and anti-inflammatory biomarkers. Similar to the results from previous trials, we did not find any effects of coffee on hs-CRP [27, 28], and TNF-α [29]. In this study, no change in NF-KB activity in PBMCs was also observed. The lack of antioxidant and anti-inflammatory responses in the present study could partly mediate the non-significant effect of supplements on liver after caffeine and / or chlorogenic acid ingestion. Although few studies in the past have attempted to assess the effect of caffeine on inflammatory biomarkers, the findings remained unclear mainly as a result of different study designs and outcomes [30]. A recent systematic review has concluded that coffee consumption was either neutrally or inversely associated with serum concentrations of inflammatory markers [30].

In a meta-analysis on cross sectional studies between 2008 and 2017, Zhang et al. showed that coffee consumption is efficient to increase adiponectin levels only in females [31]. This finding could be explained by the difference in number of fat cells between men and women [31]. In addition, intake of 4 ≤ cups of coffee in women with and without diabetes was also associated with 20% increase in adiponectin levels [32]. In contrast, Wedick et al. found increase in adiponectin levels in both genders after 8 weeks of supplementation with 5 cups of coffee/d [27]. However, in a cross sectional study on multi-ethnic Asian population, there was no relationship between coffee drinking and adiponectin concentration [33]. Our data are corroborated by the finding of this study which did not observe differences in levels of adiponectin even after including both genders. Green coffee bean extract supplementation in NAFLD patients induced significant increase in TAC and decrease in hs-CRP, but not in TNF- α [34]. Consistent with these results, a study of healthy adults who drank coffee which contained medium and high chlorogenic acid reported positive change in antioxidant capacity [35]. Our finding, however, indicate that chlorogenic acid has no major effect on antioxidant capacity in diabetic patients.

Evidence concerning the effect of regular and decaffeinated coffee on insulin sensitivity is controversial, especially as data provided by short term trials and observational studies have reached discordant results and conclusions [12]. Since 2002, many but not all observational studies have found potential beneficial effect of both caffeinated and decaffeinated coffee consumption against the incidence of developing type 2 diabetes by 30–60% in a diverse population [12, 36]. In addition, previous experimental studies have shown that chlorogenic acid is responsible for hypoglycemic effects of coffee. This result can be explained by inhibition of glucose absorption and at the cellular level, activating AMP-activated protein kinase (AMPK) [12]. However, the observational and experimental studies are not confirmed by all human short term clinical trials [37, 38].

No significant effect was found on glucose metabolism in our study, similar to previous report that evaluated the effect of coffee on this variable in type 2 diabetes [28]. We showed that chlorogenic acid may act synergistically with caffeine to increase insulin levels. This finding is consistent with a short term trial of healthy volunteers in which caffeine administrated in capsulated form (400 mg in two capsules or 870 mg in six capsules) or coffee drink (52 g coffee grounds) or roasted coffee (300 ml) [37–39]. From the result of a recent meta -analysis conducted in healthy subjects, it has been found that acute caffeine ingestion reduces insulin sensitivity [40]. However, Da Silva et al. suggested that caffeine consumption is associated with increase in insulin clearance [41]. The mechanism of action could be explained by the effects of caffein on increasing epinephrine release and free fatty acids levels or activation of adenosine receptor [27, 38]. However, tolerance to some effects of caffeine may be developed over time [27]. Moreover, caffeine stimulates insulin secretion primarily by activation of ryanodine receptors in pancreatic β-cells in a glucose dose dependent manner, with no effect at low glucose concentrations and stimulating insulin only when glucose concentration is high [42]. This mechanism may explain why caffeine has increased insulin levels in diabetic patients in our long term study.

The association between coffee consumption and serum lipids have been studied extensively, however, yielded inconsistent results [28]. A meta- analysis of clinical trials suggested possible increased serum levels of LDL-C and total cholesterol for unfiltered coffee consumption [43], but this finding was partially driven by the results of English language literature and two important trials [44, 45], while studies published before 1998 were not identified. Additional data from a meta-analysis including only randomized controlled trials involving a total of 1017 subjects provided more information about any potential effects of coffee consumption; it suggested that the serum levels of TC, LDL-C and TG may significantly increase by coffee consumption, but the effect was greater for boiled (unfiltered) coffee [46]. Although studies that used filtered coffee (removed the most of the oils by paper filter) have reported conflicting results, some studies found positive effects (decrease in cholesterol and LDL-C levels [47] and increase in HDL-C) [28], while other studies did not find any effects of coffee consumption on components of the lipid profile [48]. The preparation method of coffee can affect the concentrations of lipid altering factors such as cafestol and kahweol and caffeine [49]; however, the effects of caffeine on serum cholesterol remained unclear [46]. In one observational study, investigators have suggested that coffee consumption in a dose -dependent manner is associated with lipid-raising effect, regardless of its caffeine content [50].

Although a case-control study found an unfavorable association between caffeine consumption and cholesterol [51], one of the finding of present study was that caffeine intake (200 mg/ d) was associated with about 20 mg/ dl decrease in total cholesterol over 6 months of follow up after adjustment for potential confounders. Increase of fecal lipid extraction has been suggested as a possible mechanism underling the cholesterol –decreasing effect of caffeine [52]. In addition, in observational studies, many people do not consume coffee in isolation, but add sugar (glucose and fructose) and milk which might be responsible for increase in cholesterol levels [46].

To summarize, we did not meet the primary endpoint of showing a reduction in hepatic fat and fibrosis in NAFLD patients undergoing 6 months supplementation. Indeed, it is possible to speculate that either the period of exposure was not enough for modifying the hepatic fat and fibrosis and the biochemical indicators of hepatic involvement in NAFLD, or the link between coffee and NAFLD could be appreciated only in specific subpopulation of NAFLD patients. As we enrolled diabetic patients with the number of patients with few patients with advanced fibrosis, the results cannot be generalized to other population and would be different if non diabetic patients or NASH patients with advanced fibrosis were included in the study. Furthermore, we used the dose of 200 mg of each supplement, while some observational studies indicate that higher doses might be effective.

This study has several advantages. This is the first randomized double-blind, placebo-controlled, clinical trial that evaluated the effects of main components of coffee on patients with NAFLD and diabetes. Using the extracted components of coffee provided us an opportunity to roll out the confounding effects of coffee preparation methods, varieties in different coffee components, and variation in the amount of coffee consumption. Since our study population had a background of lower caffeine consumption (i.e. irregular coffee intake or coffee abstinence), this might resulted in more accurate findings on the studied parameters, as a tolerance to the effects of caffeine probably would not have already developed. Furthermore, we asked the patients to take supplements in the morning, which is usual mode of coffee intake. Furthermore, we combined chlorogenic acid and caffeine to determine if there could be increased benefit from adjuvant therapy. In addition, because all participants were newly diagnosed by NAFLD, they were not undertaken any treatment as a part of clinical care, which reduced the effects of confounding factors related to treatment strategies.

## Conclusion

Our study found that six months supplementation with 200 mg/day of two main components of coffee had no significant impact on non-invasive markers of hepatic steatosis and fibrosis, liver biochemistry, inflammatory and metabolic parameters in patients with NAFLD and type 2 diabetes; however, this dosage was completely safe without any reported side-effects. Further trials with higher dosages, longer duration, and different active components of coffee are highly recommended.

## Data Availability

Data are available upon reasonable request.

## Abbreviations

NAFLD: Non-alcoholic fatty liver disease
WHO: World Health Organization
CAP: Controlled attenuation parameter
TE: Transient elastography
LSM: Liver stiffness measurements
IPAQ: International physical activity questionnaire
METs: Metabolic Equivalent of Task
BIA: Body impedance analyzer
ALT: Alanine transaminase
AST: Aspartate transaminase
GGT: Gamma-glutamyl transferase
TC: Total cholesterol
HDL: High-density lipoprotein cholesterol
TG: Triglycerides
Cr: Creatinine
hs-CRP: High sensitive- C reactive protein
LDL: Low-density lipoprotein cholesterol
HbA1c: Hemoglobin A1c
TSH: Thyroid stimulating hormone
HOMA-IR: Homeostasis model assessment-estimated insulin resistance
TAC: Total antioxidant capacity
CK-18: Cytokeratin-18
PBMC: Peripheral blood mononuclear cell
TGF-β: Transforming growth factor-β
AMPK: AMP-activated protein kinase
